# KRAS/NRAS/BRAF Mutations as Potential Targets in Multiple Myeloma

**DOI:** 10.3389/fonc.2019.01137

**Published:** 2019-10-24

**Authors:** Sergiu Pasca, Ciprian Tomuleasa, Patric Teodorescu, Gabriel Ghiaur, Delia Dima, Vlad Moisoiu, Cristian Berce, Cristina Stefan, Aaron Ciechanover, Herman Einsele

**Affiliations:** ^1^Department of Hematology, Iuliu Hatieganu University of Medicine and Pharmacy, Cluj-Napoca, Romania; ^2^Department of Hematology, Ion Chiricuta Clinical Cancer Center, Cluj-Napoca, Romania; ^3^Research Center for Functional Genomics and Translational Medicine, Iuliu Hatieganu University of Medicine and Pharmacy, Cluj-Napoca, Romania; ^4^Department of Leukemia, The Sidney Kimmel Comprehensive Cancer Center at Johns Hopkins, Baltimore, MD, United States; ^5^African Organisation for Research and Training in Cancer, Cape Town, South Africa; ^6^The Rappaport Faculty of Medicine and Research Institute, Technion-Israel Institute of Technology, Haifa, Israel; ^7^Department of Internal Medicine II, University Hospital Würzburg, Würzburg, Germany

**Keywords:** multiple myeloma, KRAS, BRAF, NRAS, therapeutics

## Abstract

In multiple myeloma the mutational profile is mainly represented by translocations involving chromosome 14 and by single nucleotide mutations, frequently involving genes implicated in the mitogen activated protein kinase (MAPK) pathway, as KRAS, NRAS, and, less frequently, BRAF. Because KRAS/NRAS/BRAF mutations are associated with a higher number of mutations per patient, we hypothesize that this group of patients could benefit from therapy with checkpoint inhibitors because of the higher frequency of neo-antigens that this group would present. This might also true for IMiD therapy, because of their activatory effect on T cells. Because, KRAS/NRAS/BRAF are members of the MAPK pathway, this subgroup of patients would also benefit from inhibitors of MAPK, either directly on the specific mutation or through downstream targeting of MEK1/2 or ERK1/2 to account for a possible compensatory collateral signaling that might activate as response to upstream inhibition.

Multiple myeloma (MM) is a hematological malignancy in which the proliferating clone is represented by the plasma cells. The main clinical symptoms associated with this disease are caused by paraprotein secretion, osteoclast activation, and bone marrow involvement. The end results of the aforementioned causes are end organ damage, especially renal dysfunction, bone fractures, and pancytopenia (1). As more and more therapeutic approaches are being developed for MM, there is still no therapy offering a strong curative option for this disease (2).

The genetic changes of this malignancy are generally represented by translocations involving chromosome 14 and by single nucleotide mutations. The latter frequently involving genes implicated in the mitogen activated protein kinase (MAPK) pathway, generally represented by KRAS, NRAS, and, less commonly, by BRAF. Because of the high frequency of these mutations in MM, we hypothesize that they can be targeted either directly or downstream the activating mutation, for example at the level of MEK1/2 or ERK1/2 (3–5).

MM treatment consists mainly in the use of proteasome inhibitors (PIs), immunomodulatory drugs (IMiDs), glucocorticoids, and biological agents. Because MM is considered an incurable disease, efforts have been made to develop or to use drugs already approved for other diseases to improve the prognosis of MM (6). One such attempt is represented by the use of checkpoint inhibitors. Many of those trials were not successful either because of too low efficacy, as in the case of monotherapy or were discontinued due to toxicity, in the case of the combinations of checkpoint inhibitors with IMiDs (6). The toxicities of combining an IMiD with a checkpoint inhibitor are hypothesized to have arisen because of IMiDs effect on increasing the activation and tumor infiltration of effector T cells. This was hypothesized to improve the tumor-killing potential of T cells when associated with a checkpoint inhibitor, but only determined a raise in autoimmune complications with no improvement in efficacy. This was thought to be caused by the fact that T cells infiltrating MM are generally senescent and not exhausted, thus checkpoint inhibitors not being able to significantly enhance the activity of T cells within the MM (6, 7).

As a proof-of-concept, we analyzed the data published by Lohr et al. on MM using the cBioPortal (8–10). The inclusion criteria included patients with MM, of Caucasian race and available mutational profile. Data analysis was performed using R 3.5.3. Clustering was performed with hierarchical clustering using Euclidean distances and the complete method. Dummy coding was applied to use hierarchical clustering on dichotomial variables. Categorical variables were represented as absolute value (percent). Contingency tables were analyzed using Fisher test. Benjamini–Hochberg *p* adjustment method was used for repeated analyses. Normality of the distribution was assessed using Shapiro test and histogram visualization. Differences between two non-normally distributed groups were assessed using Wilcox test. The model used for determining the chromosomes harboring a different number of mutations between the designed groups used repeated use of t statistic with Benjamini–Hochberg adjustment. In the aforementioned model both *p*-value and adjusted *p*-value were assessed. A *p-*value under 0.05 was considered statistically significant.

Using the aforementioned criteria, we included 150 patients in the current study. Mutations present in more than 10 patients are represented in Figure 1. For a relevant coverage we selected all mutated genes that occurred in more than five patients and used them for hierarchical clustering. After removing the outliers, 116 (77.3%) patients remained with 37 (31.9%) in one cluster, and 79 (68.1%) in the other. Comparing the two clusters we observed that KRAS, NRAS, and BSN were the genes different between the two groups considering the adjusted *p*-values. Also, a different segregation of KRAS and NRAS mutations was observed, each being enriched in one of the clusters (Figure 2). Considering that clustering was highly influenced by KRAS and NRAS and the current literature on MAPK in MM (3, 4), we decided to split the initial cohort in KRAS/NRAS/BRAF mutated vs. non-mutated. Seventy-four (49.3%) patients had mutations in KRAS/NRAS/BRAF. Four (2.66%) patients had two overlapping mutations. The clinical and sample variables were not different between the two groups (Table 1). Considering the mutation data, there were a total of 5.531 distinct genes mutated between all patients. The total number of mutated genes per patient was significantly different between the two groups with more mutations in the KRAS/NRAS/BRAF group (*p* = 0.0107) (Figure 3). Considering this, we hypothesized that the higher number of mutations per patient might be caused by more frequent mutations in DNA damage repair genes. For genes implicated in DNA damage repair we used the list published by Chae et al. (11). There was no different enrichment in genes implicated in DNA damage repair between the two groups.

**Figure 1:**
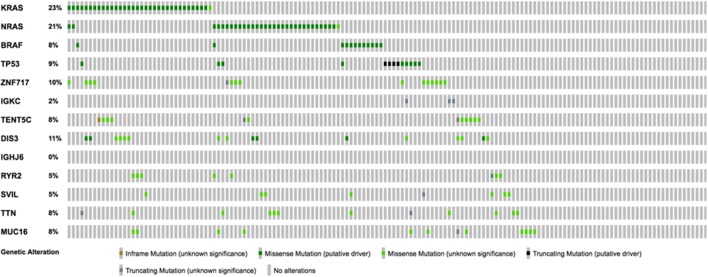
Oncoprint representation of the frequency and characteristics of genes mutated in more than 10 patients of the selected cohort.

**Figure 2:**
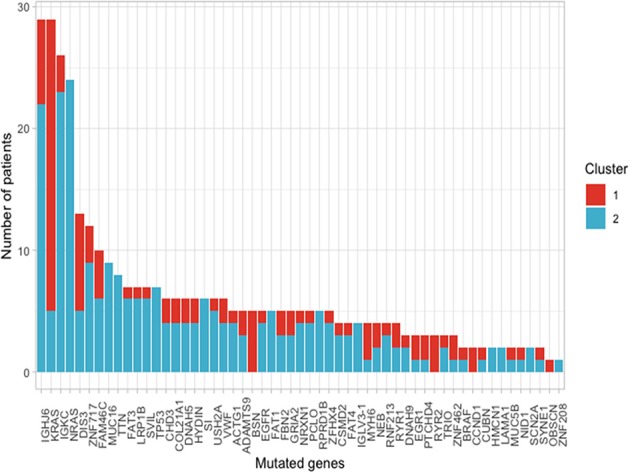
Frequency of mutations present in more than 10 patients of the selected cohort between the two clusters that resulted from using a cut with *k* = 2 of the hierarchical clustering.

**Table 1:** The clinical and sample variables were not different between the two analyzed patient cohorts.

		**Non-MAPK**	**MAPK**	***p*-value**
		***n* = 76**	***n* = 74**	
Median age; years (quartile 1, quartile 3)		63.0 (56.0, 69.0)	59.5 (53.0, 67.0)	0.151
Sex	Female	26 (34.2%)	26 (36.6%)	0.863
	Male	50 (65.8%)	45 (63.4%)	
Previous treatment	No	39 (52%)	26 (38.2%)	0.130
	Yes	36 (48%)	42 (61.8%)	
Heavy chain	A	12 (23%)	12 (24.5%)	0.816
	G	40 (77%)	36 (73.5%)	
	M	0 (0%)	1 (2%)	
Light chain	Biphenotypic	0 (0%)	1 (1.8%)	0.620
	Kappa	35 (67.3%)	41 (71.9%)	
	Lambda	17 (32.7%)	15 (26.3%)	
Hyperdiploid	No	34 (44.7%)	32 (43.2%)	0.871
	Yes	42 (55.3%)	42 (56.8%)	
Translocations	del17p13	0 (0%)	1 (4.1%)	1
	t(11;14)	14 (77.8%)	16 (66.7%)	
	t(14;16)	0 (0%)	1 (4.1%)	
	t(4;14)	4 (22.2%)	6 (25%)	

**Figure 3:**
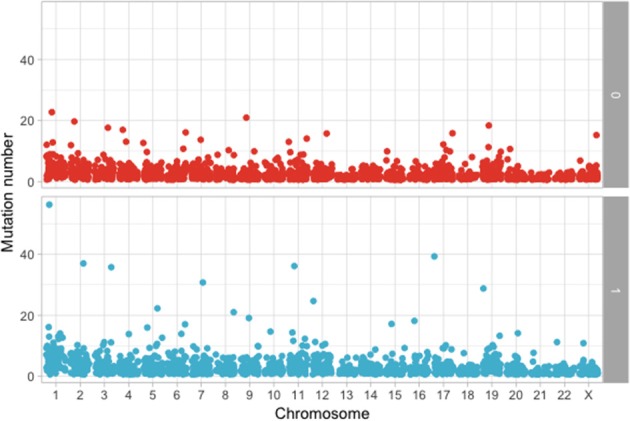
Comparison of the number of mutations between KRAS/NRAS/BRAF mutated (Yes) vs. wild type (No).

The following step was to determine if these mutations are enriched in different chromosomes between the MAPK and non-MAPK groups. Because of the low mutational count, we removed chromosome Y. Chromosomes that were different between the two groups considering the *p*-value, but not the adjusted *p*-value were chromosome 1 (*p* = 0.0153; adjusted *p* = 0.176) and 12 (*p* = 0.00416; adjusted *p* = 0.0958) (Figure 4). When adjusting for the dimension in base pairs of the chromosome, the chromosome presenting the most mutations per base-pair was represented by chromosome 19 (Figure 5). Mutated genes situated on chromosome 1 and present in more than 10 patients were NRAS, FAM46C, and RYR2, while the gene mutated in more than 10 patients from chromosome 12 was KRAS.

**Figure 4:**
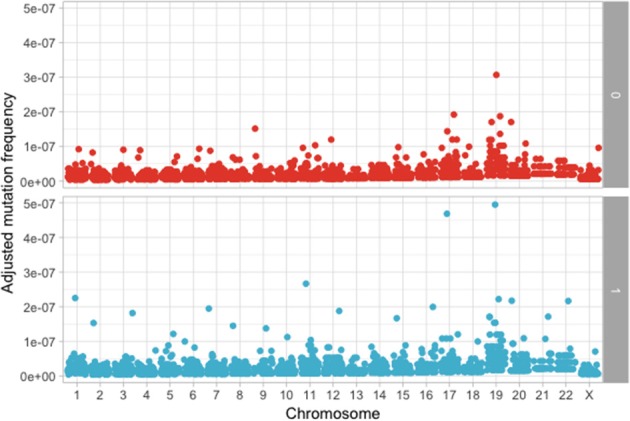
Number of mutations per chromosome between the MAPK (1) and non-MAPK (0) groups.

**Figure 5:**
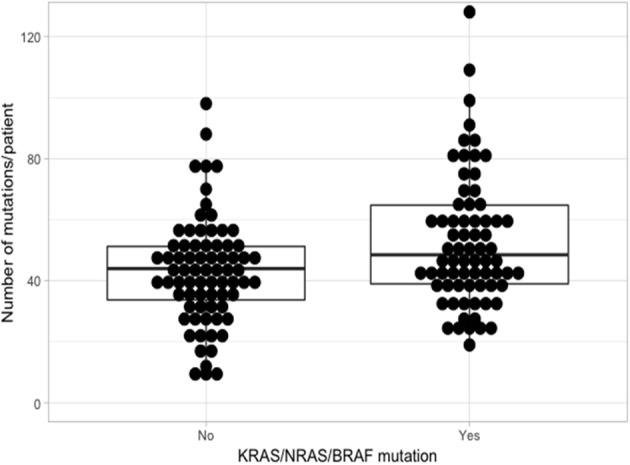
Adjusted mutation count per chromosome, calculated as number of mutations/chromosome length in base pairs between the MAPK (1) and non-MAPK (0) groups.

When considering gene mutations occurring in more than five patients, we observed that 143 (95.3%) presented any of those genes mutated. After eliminating KRAS/NRAS/BRAF from the analysis there were no differences in gene enrichment between the two groups neither in the *p*-value or adjusted *p*-value. From these results we hypothesize that the higher number of mutations in the KRAS/NRAS/BRAF group could be attributed to the selection criteria, as KRAS/NRAS/BRAF had the inclusion criteria of any three frequently mutated genes. This being said, it could still be applicable in the clinic, as we could observe the same enrichment bias when selecting for KRAS/NRAS/BRAF mutated MM.

Because KRAS/NRAS/BRAF mutations are associated with a higher number of mutations per patient, we hypothesize that this group of patients could benefit from therapy with checkpoint inhibitors because of the higher frequency of neo-antigens that this group would present. This might also true for IMiD therapy, because of their activatory effect on T cells. Because, KRAS/NRAS/BRAF are members of the MAPK pathway, this subgroup of patients would also benefit from inhibitors of MAPK, either directly on the specific mutation or through downstream targeting of MEK1/2 or ERK1/2 to account for a possible compensatory collateral signaling that might activate as response to upstream inhibition (3–5). Moreover, because of the known efficacy of IMiDs in MM (12), we would rather consider the combination of MAPK inhibitors with an IMiD, rather than a checkpoint inhibitor.

The major limitation of the study is that it presents a clinical comment, not experimental data validated by a large patient cohort. Thus, even if it is plausible and it may have deep clinical impact, the manuscript should be regarded at this point as a hypothesis, that need further extensive validation or invalidation on a large population cohort.

Still, the current hypothesis raises several important questions regarding the mutational burden in the MAP kinase pathways and should be regarded as a steppingstone toward a curative approach for MM. Two questions in particular remain to be answered at the end of this letter: Should we start targeting MAPK pathway in MM in the cases where it is activated through KRAS/NRAS/BRAF mutations? Would these inhibitors present an additive or synergistic effect when associated with checkpoint inhibitors or with IMiDs? This letter should stand as a hypothesis-generating paper as the clinically relevant answer to the aforementioned questions will most likely reside in clinical trials on multiple myeloma focused on MAPK inhibitors and/or checkpoint inhibitors.

## Data Availability Statement

All datasets generated for this study are included in the manuscript/supplementary files.

## Ethics Statement

The manuscript presents a TCGA-based bioinformatics analysis, with no patient data presented. The TCGA analysis is already published and presented online. Therefore, approval and consent were not required.

## Author Contributions

All authors contributed in the conceptualization of the data, as well as in data analysis. SP and CT write the manuscript. AC and HE supervised the work.

### Conflict of Interest

The authors declare that the research was conducted in the absence of any commercial or financial relationships that could be construed as a potential conflict of interest.
